# Genome-wide screening in the haploid system reveals *Slc25a43* as a target gene of oxidative toxicity

**DOI:** 10.1038/s41419-022-04738-4

**Published:** 2022-03-30

**Authors:** Jinxin Zhang, Yiding Zhao, Yaru Tian, Mengyang Geng, Yan Liu, Wenhao Zhang, Ling Shuai

**Affiliations:** 1 State Key Laboratory of Medicinal Chemical Biology and College of Pharmacy, Nankai University, Tianjin, 300350 China; 2 Department of Obstetrics, Tianjin First Central Hospital, Nankai University, Tianjin, 300192 China; 3 Chongqing Key Laboratory of Human Embryo Engineering, Chongqing Health Center for Women and Children, Chongqing, 400013 China; 4 Tianjin Central Hospital of Gynecology Obstetrics/Tianjin Key Laboratory of Human Development and Reproductive Regulation, Tianjin, 300052 China

**Keywords:** Cell death, Stem cells, Screening, Target identification

## Abstract

Reactive oxygen species (ROS) are extensively assessed in physiological and pathological studies; however, the genes and mechanisms involved in antioxidant reactions are elusive. To address this knowledge gap, we used a forward genetic approach with mouse haploid embryonic stem cells (haESCs) to generate high-throughput mutant libraries, from which numerous oxidative stress-targeting genes were screened out. We performed proof-of-concept experiments to validate the potential inserted genes. *Slc25a43* (one of the candidates) knockout (KO) ESCs presented reduced damage caused by ROS and higher cell viability when exposed to H_2_O_2_. Subsequently, ROS production and mitochondrial function analysis also confirmed that *Slc25a43* was a main target gene of oxidative toxicity. In addition, we identified that KO of *Slc25a43* activated mitochondria-related genes including *Nlrx1* to protect ESCs from oxidative damage. Overall, our findings facilitated revealing target genes of oxidative stress and shed lights on the mechanism underlying oxidative death.

## Introduction

Redox metabolism is a critical metabolic pathway in diverse biological processes, such as organism development, homeostasis and diseases. Redox metabolism includes the removal or production of reactive oxygen species (ROS) (e.g., hydrogen peroxide (H_2_O_2_), superoxide (O_2_^−^) and hydroxyl radical (HO·)) [1, 2]. A moderate increase in ROS can promote cell proliferation, whereas excessive ROS production can activate oxidative stress. Oxidative stress occurs when there is an imbalance between the generation and scavenging of free radicals and induces oxidative damage to lipids, proteins, DNA and genomic stability [3–5]. Therefore, maintaining ROS homeostasis is crucial for normal cell growth and survival. Furthermore, ROS-induced oxidative stress is likely responsible for the onset and progression of several diseases [6]. Emerging evidence shows that cancer cells, especially those in advanced tumors, frequently exhibit high oxidative stress [7]. Pre-treatment of highly metastatic tumor cells with ROS scavengers suppresses their metastatic potential in mice [8]. Therefore, understanding ROS regulatory mechanisms might have potential value in clinical research [9].

Many efforts have been made to clarify the mechanism underlying ROS-induced oxidative stress in the context of cell death. Different types of ROS inducers have distinct diffusion capabilities and diverse effects on cells. H_2_O_2_ easily penetrates the cell membrane, which makes it an ideal inducer of oxidative stress in cells [10]. Treatment with high concentrations of H_2_O_2_ induces severe cell death through apoptosis [11], autophagy [12] and oxeiptosis [13]. Excessive H_2_O_2_ results in cytotoxicity by directly attacking cells at the molecular level or indirectly by generating secondary reactive species such as HO· [14]. Although the intrinsic antioxidant mechanisms in cells have been widely studied recently, it is still unclear which genes are involved in oxidative stress.

Mammalian haploid cell lines are powerful tools for genetic screening to uncover unknown gene functions, owing to their single genome feature [15]. Not only mouse haESCs have advantages to discover *Gpr107* as a targeting gene for ricin toxicity [16], but also haploid KBM7 cells are advanced to figure out crucial genes (including *Acsl4*) for ferroptosis [17].

Since haploid systems work well for metabolism- and disease-related genetic screening, the concept of discovering target genes of oxidative stress response mechanisms based on these systems is interesting. In this study, using the *piggyBac* (PB) transposon-mediated method, we introduced high-throughput mutations in mouse haESCs and conducted genetic screening for oxidative toxicity, revealing numerous insertions related to the oxidative stress response. Furthermore, we determined whether the KO of candidate genes could protect ESCs from oxidative stress and discussed the potential underlying mechanism.

## Materials and methods

### Cell culture

The mouse haESCs used were established by our group, as previously reported [18]. The ESC medium consisted of DMEM/F12 (Thermo, 12500062, Grand Island, USA) supplemented with 10% KOSR (Thermo, A3181502), 7% fetal bovine serum (BI, 04-002-IACS, Kibbutz Beit-Haemek, Israel), 1% nonessential amino acids (Thermo, 11140050), 0.1 mM β-mercaptoethanol (Thermo, 21985023), 100 μg/ml penicillin-streptomycin (Thermo, 15140122), 1000 U/ml leukemia inhibitory factor (Sino biological; Beijing, 50756-MNAH), 1 μM PD0325901 (MCE, HY10254, Shanghai, China) and 3 μM CHIR99021 (MCE, HY10182). ES cells were passaged with 0.25% trypsin-EDTA (Thermo, 25200072) every 2 days. In the H_2_O_2_ (Alfa, L14000, Ward Hill, USA) treatment experiment, cells were cultured in ESC medium supplemented with H_2_O_2_, and β-mercaptoethanol was withdrawn. All the cell cultures were tested for mycoplasma free by PCR with specific primers weekly.

### Purification of haploid cells by FACS and chromosome spread analysis

To purify haploid cells, dissociated cells were incubated with 5 μg/ml Hoechst 33342 (Thermo, H3570) in a water bath at 37 °C for 30 min and sorted on a MoFlo XDP (Beckman, CA, USA) cell sorter. Diploid ES cells were used as a control.

For chromosome spread analysis, ESCs (haESCs and WT-diploid ESCs (WT-diESCs)) were incubated with 0.2 μg/ml nocodazole (MCE, HY-13520) for 12 h. Cells were trypsinized to generate single cells and resuspended in 75 mM KCl at 37 °C for 30 min. Samples were fixed in methanol:acetic acid (3:1 in volume) for 30 min and dropped onto precooled slides. Then, the cells were stained with Giemsa (Sigma, GS500) solution for 7 min before observation.

### Plasmid construction and cell transfection

The PB-trapping vector was constructed according to a previous report [19]. To construct the *Slc25a43* knockout (KO) plasmids, the pSpCas9 (BB)-2A-GFP (PX458) vectors were digested and dephosphorylated by BbsI (Thermo, FD1014) and FastAP (Thermo, EF0654). *Slc25a43*-targeting sgRNAs were designed using the CRISPR design tool (http://crispr.mit.edu). Single-strand oligonucleotides were synthesized by a local company. Each pair of oligonucleotides was annealed and ligated into a lined PX458 vector with T4PNK (Takara, 2021A, Kusatsu, Japan). All plasmids were purchased from Addgene (MA, USA). All the primers used were listed in Table S1.

For the gene-trapping experiment, 2 × 10^6^ haESCs were transfected with 3 μg of PBase plasmid and 9 μg of PB-trapping plasmid using the LTX kit (Thermo, 15338100) according to the manufacturer’s instructions. Then, the cells were selected via incubation with 1 μg/ml puromycin (Thermo, A1113803) for 48 h and further screened for genes targeting oxidative toxicity. Splinkerrette PCR was utilized to identify the insertion sites after PB transfection as previously described [20]. Splinkerrette PCR products of ML1 and ML2 were sent to a local company (Novogene) for next-generation sequencing. The bioinformatics analysis of insertions was conducted according to a previous report [21].

To obtain *Slc25a43*-KO cells, ~2 × 10^6^ WT-diESCs were transfected with 6 μg sgRNA-1 and 6 μg sgRNA-2 using the LTX kit. GFP-positive cells were sorted 48 h after transfection by a MoFlo XDP. Subclones were randomly picked for genotyping.

### Quantitative PCR and western blotting

Total RNA was extracted using the TRIzol (Thermo, 15596018) method. cDNA was obtained using Hifair II 1st Strand cDNA Synthesis SuperMix for qPCR (Yeasen, 11123ES60, Shanghai, China). Quantitative PCR was performed with SYBR Green reagents (Yeasen, 11202ES03). All the primers used were listed in Table S1.

Protein samples were extracted using RIPA lysis solution (Solarbio, R0020, Beijing, China) following the manufacturer’s protocol. Lysates were centrifuged for 5 min at 4 °C. Equal amounts of cell lysates were separated by SDS–PAGE for Western blotting. The primary antibodies used were anti-SLC25A43 (ABclonal, A10726, Wuhan, China) and anti-GAPDH (Santa, sc-365062, Texas, USA). The secondary antibodies were goat anti-rabbit IgG (H + L)-HRP (Sungene, LK2001, Tianjin, China) and goat anti-mouse IgG (H + L)-HRP (Sungene, LK2003).

### Cell viability assays

Cell viability was assessed in 96-well plates using Cell Counting Kit-8 (Yeasen, 40203ES76). Cells were treated with 0 mM, 0.4 mM, 0.8 mM, and 1.2 mM H_2_O_2_ for 0, 0.25, 4, and 8 h, respectively. Cell viability was reported as a percentage relative to the negative control.

### Detection of ROS production and mitochondrial membrane potential

To detect ROS production, 1 × 10^5^ cells were seeded in each well (a 12-well plate) 1 day before the experiment. Cells were treated with 0.8 mM H_2_O_2_ for 0.8 mM H_2_O_2_ for 0, 2, 4 and 8 h, separately. Treated cells were trypsinized to single cells and resuspended in 500 μl PBS for subsequent detection. The samples were supplemented with 25 μM H_2_DCFDA (Sigma, D6882, USA), 2 μM C11-BODIPY^581/591^ (Thermo, D3861) and 5 μM MitoSOX (Thermo, M36008), respectively. For detection of mitochondrial membrane potential (MMP) and cytosolic ROS, cells were treated with 0.8 mM H_2_O_2_ for 4 h, trypsinized to generate single cells, and resuspended in 2 μg/ml rhodamine 123 (Thermo, R302) and 5 μM CellROX Orange Reagent (Yeasen, 50103ES50), respectively. After incubation for 20 min at 37 °C, the cells were centrifuged, resuspended and analyzed using a flow cytometer. All fluorescence-activated cell sorting (FACS) data were analyzed using FlowJo software (San Carlos, USA).

### Immunofluorescence image capturing of live cells

Cells costained with 5 μM MitoSOX and 5 μg/ml Hoechst 33342 were treated with 0.8 mM H_2_O_2_, and images of the cells were captured by confocal microscopy (Leica TCS SP8, Germany) at 0, 1, 2, 3 and 4 h. In another parallel experiment, H_2_O_2_-treated cells (0.8 mM, 4 h) were costained with 50 nM MitoTracker Red CMXRos (Beyotime, C1035, Shanghai, China) and 5 μg/ml Hoechst 33342 for imaging capture.

### Detection of ATP, GSH and SOD

ATP, GSH and SOD were measured using an ATP Assay Kit (Beyotime, S0026), a GSH Assay Kit (Beyotime, S0053) and a Total Superoxide Dismutase Assay Kit (Beyotime, S0101M), respectively, according to the manufacturer’s protocols.

### In vivo study

Severe combined immunodeficient (SCID) mice were purchased from Beijing Vital River Laboratory Animal Technology Co., Ltd. All animal experiments were performed according to the ethical guidelines of the Nankai University Animal Centre.

Teratomas were established by subcutaneous injection of *Slc25a43*-KO ESCs and WT-diESCs (2 × 10^7^ cells for each mouse) into SCID mice. Randomization and single blinding applied during data collection, and the volume of the teratomas was calculated using the formula (length × width^2^)/2. After the teratomas formed, the mice were intratumorally injected with H_2_O_2_ (at 0, 0.25, 0.5 and 0.1% concentrations (w/w)). Each injection was performed every 2 days. After seven injections, the teratomas were imaged, removed and weighed.

### RNA-seq analysis

All the RNA-seq data were sequenced by a local company (Novogene). The abundance of transcripts was counted by Kallisto with Gencode M18 and further summarized for each gene by the R package tximport. Filtered clean and sound genes were normalized using relative log-expression from DESeq2. The heatmap R function was utilized to describe the differentially expressed genes (DEGs), and ggplot2 (volcano plot) was utilized to visualize the DEGs.

## Results

### Mutant mouse haESCs with resistance to oxidative toxicity are screened out

**Fig. 1:**
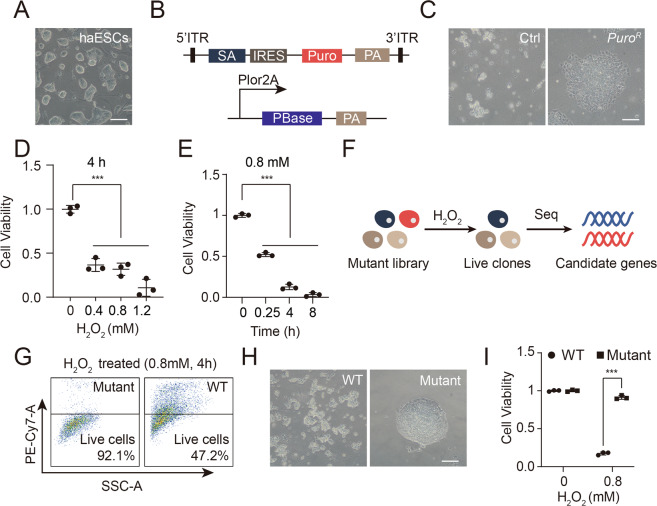
High-throughput genetic screening to identify genes sensitive to H_2_O_2_. **A** Phase-contrast image of mouse haESCs used for screening. Scale bar, 100 μm. **B** Vector designs of the PB-trapping vector and PBase. **C** Surviving PB-trapping vector-transfected haESC colonies after selection by puromycin for 48 h. HaESCs without transfection were used as controls. Scale bar, 100 μm. **D** Cell viability of WT-haESCs treated with 0, 0.4, 0.8 and 1.2 mM H_2_O_2_ for 4 h. Data represent three independent experiments. *t-*test, ***p < 0.001. Data are presented as the mean ± SD. **E** Cell viability of haESCs treated with 0.8 mM H_2_O_2_ for 0, 0.25, 4, and 8 h. Data represented three independent experiments. *t-*test, ***p < 0.01, ***p < 0.001. Data were presented as the mean ± SD. **F** Schematic overview of the identification of H_2_O_2_ toxicity-targeting genes. Surviving cells were analyzed by subsequent sequencing. **G** DRAQ7 staining analysis of cell viability in Puro^r^ haESCs and WT-haESCs treated with 0.8 mM H_2_O_2_ for 4 h. PE-Cy7-positive cells indicated dead cells in the cell cultures. **H** Phase-contrast images of WT-haESCs (left) and Puro^r^ haESCs (right) 4 days after being treated with 0.8 mM H_2_O_2_ for 4 h. Scale bar, 100 μm. **I** Cell viability analysis of Puro^r^ haESCs and WT-haESCs 4 days after treatment with 0.8 mM H_2_O_2_ for 4 h by CCK-8 assay. Data represented three independent experiments. *t*-test, ***p < 0.001. Data were presented as the mean ± SD.

To generate genome-wide mutant libraries, we used mouse haESCs to perform gene trapping with the designed PB-trapping vectors. The haESCs used in this study were derived from our group as reported previously, and the genetic background of the haESCs was 129 Sv/Jae [18]. These haESCs showed standard domed mouse ESC colonies (Fig. 1A). After several rounds of enrichment for haploid cells with FACS using Hoechst 33342, haESCs with a high proportion of haploid cells (Fig. S1A) were utilized to generate homozygous mutants on the whole genome scale. The chromosome spread analysis results further confirmed the haploidy in the cell cultures (Fig. S1B). Given the high transposition activity and low bias toward the genome of the PB transposon [22–24], we used a PB-trapping vector carrying a puromycin resistance (Puro^r^) gene to allow for massive insertions (Fig. 1B). Approximately 2 × 10^6^ haESCs were transfected with the PB-trapping vector and selected by 1 μg/ml puromycin. Two days later, some of the transfected haESCs survived, whereas most of the cells in the control group died (Fig. 1C). This result indicated that these Puro^r^ haESCs were efficiently inserted with the PB-trapping vector.

H_2_O_2_ is a common type of oxidant, and a certain concentration of H_2_O_2_ can induce severe oxidative toxicity and cause cell death in cell cultures [25]. In this regard, we chose H_2_O_2_ as an oxidant to investigate genes related to oxidative stress. To detect the optimal lethal dosage of H_2_O_2_, we treated WT-haESCs with 0, 0.4, 0.8 and 1.2 mM H_2_O_2_ for 4 h independently. The CCK-8 assay demonstrated that once H_2_O_2_ was added, haESCs exhibited significantly reduced cell viability (Fig. 1D). The cells recovered when H_2_O_2_ was withdrawn in the 0.4 mM group (data not shown). However, none of the cell cultures escaped cell death when the dose of H_2_O_2_ was over 0.8 mM. Herein, we defined 0.8 mM as the optimal concentration of H_2_O_2_ for the subsequent selection experiment. We treated haESCs with 0.8 mM H_2_O_2_ for different times (0.25, 4 and 8 h) to find an optimal treatment time compared to the 0 h group. The viability of haESCs showed a significant reduction in the different treatment groups (Fig. 1E). None of the cells could survive when the H_2_O_2_ treatment time was over 4 h, which was why we defined this duration as our treatment time. Thereafter, we designed a strategy to perform genetic screening of H_2_O_2_-resistant genes (Fig. 1F). Briefly, PB-trapped haESCs were treated with 0.8 mM H_2_O_2_ for 4 h, and the surviving cells were collected for sequencing. The DRAQ7 staining analysis results indicated that the PB-trapped haESCs showed higher viability than the WT-haESCs after treatment with H_2_O_2_ (Fig. 1G). Approximately 4 days later, some PB-trapped colonies survived, whereas the WT-haESCs hardly showed any live cells (Fig. 1H). The CCK-8 assay further confirmed this result (Fig. 1I). To assess the insertion efficiency, we randomly picked 17 subclones for the analysis of insertion sites by inverse PCR. The results demonstrated that all subclones carried insertions (Fig. S1C). The Sanger sequencing results suggested that some of the PB vectors could integrate into the gene body (Fig. S1D).

### Inserted genes related to oxidative stress are revealed

**Fig. 2:**
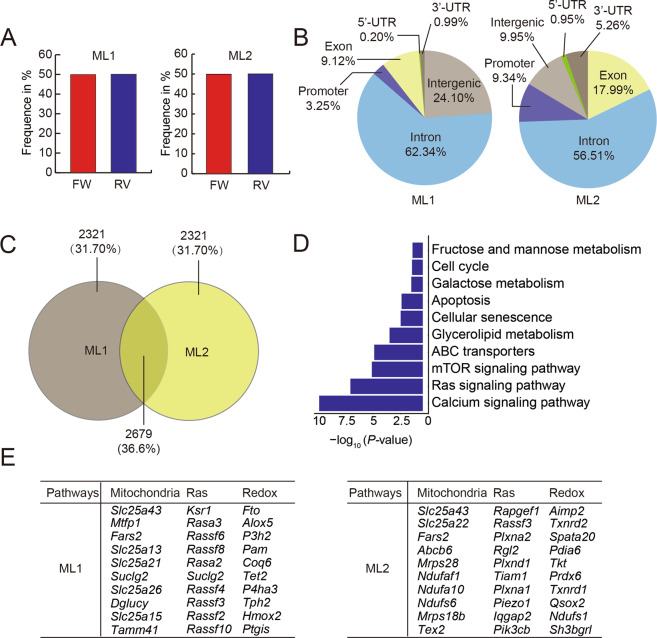
Bioinformatics analysis of integrations in HaESCs. **A** Proportion of the insertional orientation (sense/antisense) after PB integration in mutant library 1 (ML1) and mutant library 2 (ML2). **B** Analysis of integration sites after H_2_O_2_ selection over genomic regions: promoters (1 kb upstream of the transcription starting sites), intragenic regions and intergenic regions in ML1 and ML2. **C** Venn diagram illustrating the overlapping genes among the top 5000 genes in ML1 and ML2. **D** KEGG analysis of the overlapping genes of ML1 and ML2. **E** Lists of insertion genes from H_2_O_2_-resistant screening related to mitochondria, Ras signaling pathway and redox metabolism.

Next, we repeated the genetic screening experiments and screened two oxidative toxicity-targeting gene libraries independently. The results showed that multiple insertions were amplified in each library (mutant library 1 (ML1) and mutant library 2 (ML2)) by Splinkerrette PCR products (Fig. S2A). According to deep sequencing, there were ~22 million independent insertions covering more than 20,000 genes in the two oxidative toxicity-targeting gene libraries. Both libraries had half of the insertions integrated into the sense orientation, while the other half integrated into the antisense orientation (Fig. 2A). In addition, ~24.10% of insertions were located in intergenic regions, and 75.90% of the insertions were integrated in intragenic regions (including coding regions, promoter regions, introns and 5′-UTRs and 3′-UTRs) in ML1. The insertions in ML2 also covered the whole genome (9.95% intergenic regions and 90.05% intragenic regions, respectively) without bias (Fig. 2B). There were 2679 overlapping inserted genes between ML1 and ML2 among the top 5000 genes (Fig. 2C). Kyoto Encyclopedia of Genes and Genomes analysis of the overlapping inserted genes of ML1 and ML2 illustrated that insertions enriched for calcium signaling and Ras signaling were the top 2 pathways (Fig. 2D). The calcium signaling pathway coordinates protein synthesis, mitochondrial activity and cell proliferation [26, 27], which is involved in oxidative stress. In addition, Ras signaling is related to antioxidant capacity [28]. Gene ontology (GO) analysis data indicated that most of the inserted genes were correlated with membrane transport, mitochondria and redox (Fig. S2B). The inserted genes of both libraries related to mitochondrial function, Ras and redox metabolism were shown (Fig. 2E, F).

### Knockout of *Slc25a43* enhanced the oxidative stress resistance of ESCs

**Fig. 3:**
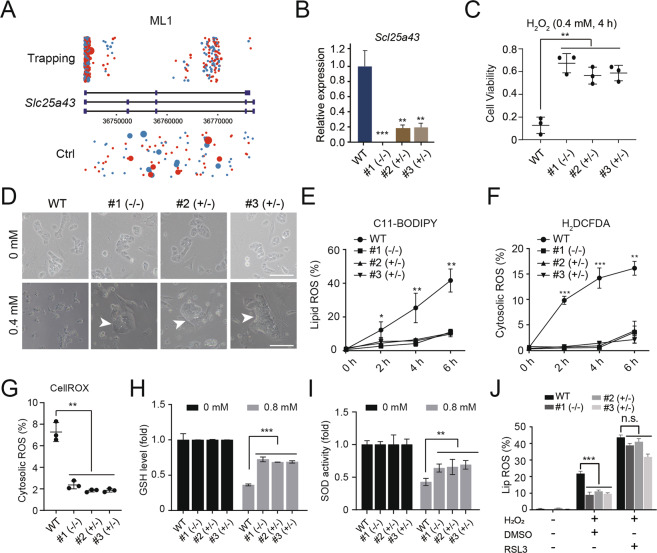
Validation of *Slc25a43* in the oxidative toxicity resistance assay. **A** Sense (red) and antisense (blue) insertions of *Slc25a43* in ML1. The rectangles indicate the exons, and the size of the circle indicates the insertion number. **B** The expression levels of *Slc25a43* in #1 (−/−), #2 (+/−), #3 (+/−) and WT-diESCs by qPCR. Data represented three independent experiments. *t-*test, **p < 0. 01, ***p < 0.001. Data were presented as the mean ± SD. **C** Cell viability of #1 (−/−), #2 (+/−), #3 (+/−) and WT-diESCs treated with 0.4 mM H_2_O_2_ for 4 h. Data represented three independent experiments. *t-*test, **p < 0.01. Data were presented as the mean ± SD. **D** Phase-contrast images of #1 (−/−), #2 (+/−), #3 (+/−) and WT-diESCs 4 days after being treated with 0.4 mM H_2_O_2_ for 4 h. Scale bar, 100 μm. **E** Lipid ROS production in *Slc25a43*-KO ESCs and WT-diESCs treated with 0.8 mM H_2_O_2_ for 0, 2, 4 and 6 h by flow cytometry analysis with C11-BODIPY staining. Data represented three independent experiments. *t-*test, *p < 0.05, **p < 0.01. Data were presented as the mean ± SD. **F** Cytosolic ROS levels in *Slc25a43*-KO ESCs and WT-diESCs treated with 0.8 mM H_2_O_2_ for 0, 2, 4 and 6 h by flow cytometry analysis with H_2_DCFDA staining. Data represent three independent experiments. *t-*test, **p < 0.01, ***p < 0.001. Data are presented as the mean ± SD. **G** Cytosolic ROS levels in *Slc25a43*-KO ESCs and WT-diESCs treated with 0.8 mM H_2_O_2_ for 4 h by flow cytometry analysis with CellROX staining. Data represented three independent experiments. *t-*test, **p < 0.01. Data are presented as the mean ± SD. **H** GSH levels in *Slc25a43*-KO ESCs and WT-diESCs treated with/without 0.8 mM H_2_O_2_ for 4 h. Data represented three independent experiments. *t-*test, **p < 0.01, ***p < 0.001. Data were presented as the mean ± SD. **I** SOD activities in *Slc25a43*-KO ESCs and WT-diESCs treated with/without 0.8 mM H_2_O_2_ for 4 h. Data represented three independent experiments. *t-*test, **p < 0.01. Data are presented as the mean ± SD. **J** Lipid ROS production in *Slc25a43*-KO ESCs and WT-diESCs treated with 0.8 mM H_2_O_2_ in the presence or absence of RSL3 (1 μM) for 4 h by flow cytometric analysis with C11-BODIPY staining. Data represented three independent experiments. *t-*test, ***p < 0.001. Data were presented as the mean ± SD.

Mitochondria play very important roles in oxidative stress [10, 29]. Interestingly, most of our insertions were mitochondrial carriers. One of the mitochondrial carriers, solute carrier family 25 member 43 (*Slc25a43*), occurred in both ML1 and ML2 and was chosen for subsequent validation experiments. There were more independent insertions located at *Slc25a43* in MLs (ML1 and ML2) than in the control group (non-H_2_O_2_ screened) and were enriched in exons (Figs. 3A and S3A). Thereafter, we investigated whether *Slc25a43*-null could enable the antioxidant ability of the cells. We designed two Cas9-mediated guide RNA (gRNA) vectors to induce *Slc25a43* KO in WT-diESCs (Fig. S3B). To enrich Cas9-GFP-positive ESCs, ~11.3% of GFP-positive cells were sorted and further cultured for subclone selection (Fig. S3C). Next, 24 subclones were randomly picked and expanded for genotyping. The results showed that three subclones (#1 (−/−), #2 (+/−) and #3 (+/−)) carried different KO status in the *Slc25a43* gene (Fig. S3D). The expression of *Slc25a43* in the three subclones was reduced significantly, as indicated by qPCR (Fig. 3B). Western blotting further confirmed that SLC25A43 protein levels were decreased in all three subclones (Fig. S3E). To test whether KO of *Slc25a43* might enhance the antioxidant capacity of mouse ESCs, we treated #1 (−/−), #2 (+/−), #3 (+/−) and WT-diESCs with 0.4 mM H_2_O_2_ for 4 h independently. The CCK-8 assay results indicated that the cell viability of *Slc25a43*-KO lines was higher than that of WT-diESCs after treatment with H_2_O_2_ (Fig. 3C). All the *Slc25a43*-KO lines had live colonies 2 days after H_2_O_2_ treatment, whereas the control WT-diESCs hardly had any surviving cells (Fig. 3D). Oxidant stimuli usually have effects on ROS production [30], which results in tremendous harm to cells. Therefore, we detected the ROS levels in *Slc25a43*-null cells after H_2_O_2_ treatment and compared them with those in WT-diESCs. We stained cells with C11-BODIPY and found that lipid ROS levels in *Slc25a43*-null ESCs were lower than those in WT-diESCs, suggesting that KO of *Slc25a43* could prevent lipid peroxidation induced by H_2_O_2_ (Fig. 3E and Table S2). Similarly, cytosolic ROS increase was suppressed in the *Slc25a43*-null ESCs compared with that in WT-diESCs according to H_2_DCFDA staining (Fig. 3F and Table S2). To further verify that KO of *Slc25a43* can protect ESCs against H_2_O_2_-induced oxidative damage, we assessed cytosolic ROS production with the optimized probe CellROX. Compared to WT-diESCs, *Slc25a43*-null ESCs displayed a significant reduction in H_2_O_2_-induced intracellular ROS production (Fig. 3G). To explore why the *Slc25a43*-null ESCs presented better cell viability than WT-diESCs treated with H_2_O_2_, we assessed the major antioxidants (reduced glutathione (GSH) and superoxide dismutase (SOD) [31]) in these cell cultures. It was reported that the absence of GSH or SOD increased oxidative damage to the cells [25, 32, 33]. We found that deletion of *Slc25a43* maintained the GSH levels and SOD activities in cells treated with H_2_O_2_ (Fig. 3H, I). Overall, the *Slc25a43*-KO cells had a stronger antioxidant capacity when exposed to H_2_O_2_ than WT-diESCs.

We next investigated the mechanism by which *Slc25a43*-KO regulates oxidative stress resistance. GPX4 is a phospholipid hydroperoxidase that combines with GSH to eliminate lipid peroxides [34]. Inactivation of GPX4 with the inhibitor RSL3 triggers lipid peroxidation and results in cell death [35]. Based on these findings, we treated the cells with the inhibitor RSL3 and found that ROS production in *Slc25a43*-KO ESCs increased significantly when RSL3 was added (Fig. 3J). This result suggested that *Slc25a43* participates in redox regulation as part of a potential mechanism of regulating lipid peroxidation associated with GPX4.

### *Slc25a43*-null ESCs maintain mitochondrial function in the presence of H_2_O_2_

**Fig. 4:**
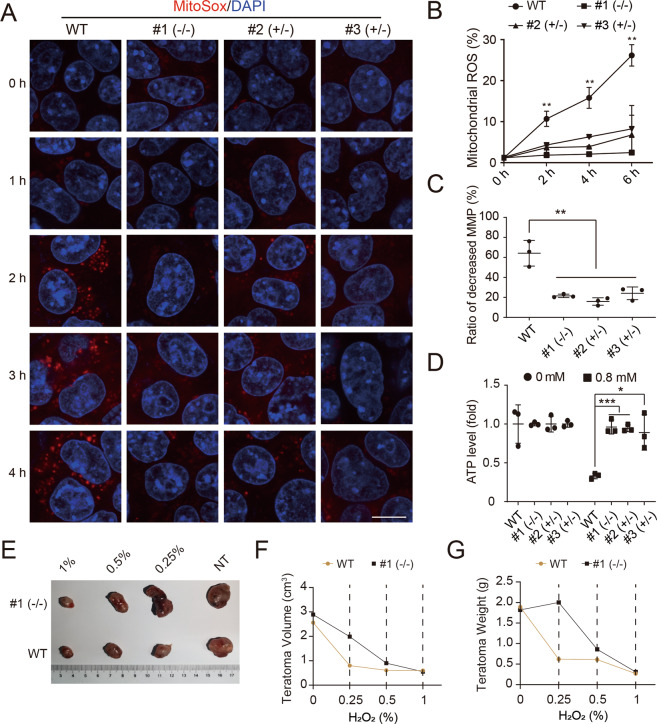
*Slc25a43*-null inhibited H_2_O_2_-triggered oxidative damage by protecting mitochondrial function. **A** Live-cell imaging of mitochondrial ROS levels by MitoSOX staining. Scale bar, 50 μm. **B** Flow cytometric analysis of mitochondrial ROS levels in *Slc25a43*-KO ESCs and WT-diESCs (samples were treated with 0.8 mM H_2_O_2_ for 0, 2, 4 and 6 h). Data represented three independent experiments. *t-*test, **p < 0.01. Data were presented as the mean ± SD. **C** Detection of MMP in *Slc25a43*-KO ESCs and WT-diESCs treated with H_2_O_2_ (0.8 mM, 4 h) by FACS analysis using rhodamine 123 staining. Data represented three independent experiments. *t-*test, *p < 0.05, **p < 0.01, ***p < 0.001. Data were presented as the mean ± SD. **D** ATP levels were detected in *Slc25a43*-KO ESCs and WT-diESCs treated with/without 0.8 mM H_2_O_2_ for 4 h. Data represent three independent experiments. *t-*test, ***p < 0.001. Data are presented as the mean ± SD. **E** The image of teratomas derived from *Slc25a43*-KO ESCs and WT-diESCs treated with 0, 0.25, 0.5 and 1% H_2_O_2_ for 14 days. **F** The volume of teratomas from SCID mice were compared between *Slc25a43*-KO and WT groups. **G** The weight of teratomas from SCID mice were compared between *Slc25a43*-KO and WT groups.

Given that *Slc25a43* KO induced H_2_O_2_ resistance in ESCs, it was quite important to address the potential underlying mechanisms. Mitochondria play a key role in redox metabolism, and the resulting damage could promote ROS production and impair cellular function [36]. Next, we investigated whether KO of *Slc25a43* could affect the function of mitochondria treated with H_2_O_2_. We found that *Slc25a43*-KO ESCs showed less positive MitoSox signals than WT-diESCs after H_2_O_2_ treatment (Fig. 4A), which was further confirmed by flow cytometric analysis (Fig. 4B and Table S2). Previous reports showed that damaged mitochondria showed weaker fluorescence intensity because of collapsing of inner membrane potential [37]. The collapse of MMP tended to trigger cell death [38]. *Slc25a43*-null cells showed stronger fluorescence intensity than WT-diESCs treated with H_2_O_2_ using MitoTracker Red CMSRos, a fluorescent dye dependent on the MMP (Fig. S4A). The results of rhodamine 123 staining indicated that *Slc25a43*-KO ESCs showed higher MMP than WT-ESCs after H_2_O_2_ treatment (Fig. 4C). The prominent function of mitochondria was the production of ATP for cells via respiration. Thus, we tested the production of ATP in *Slc25a43*-KO ESCs and WT-diESCs treated with H_2_O_2_. The results showed that the concentrations of ATP produced in *Slc25a43*-KO ESCs were higher than those in WT-diESCs treated with H_2_O_2_ (Fig. 4D). These data proved that H_2_O_2_ treatment preserved mitochondrial function in *Slc25a43*-KO ESCs. To investigate whether *Slc25a43* KO enhanced the antioxidant capacity of ESCs in vivo, we subcutaneously injected *Slc25a43*-KO ESCs and WT-diESCs separately into SCID mice. All 2-week-old teratomas were injected with 0%, 0.25%, 0.5% and 1% concentrations of H_2_O_2_ separately for another 2 weeks. The results showed that there were significant differences in size, volume and weight between the *Slc25a43*-KO ESCs and WT-diESCs treated with 0.25% H_2_O_2_ (Fig. 4E–G). However, there were no obvious differences between the two groups when treated with 0.5% and 1% H_2_O_2_, suggesting that *Slc25a43* KO promoted the antioxidant capacity of ESCs at specific physiological concentrations in vivo.

### Knockout of *Slc25a43* activates some mitochondrial-related genes, including *Nlrx1*, to gain resistance to oxidative stress

**Fig. 5:**
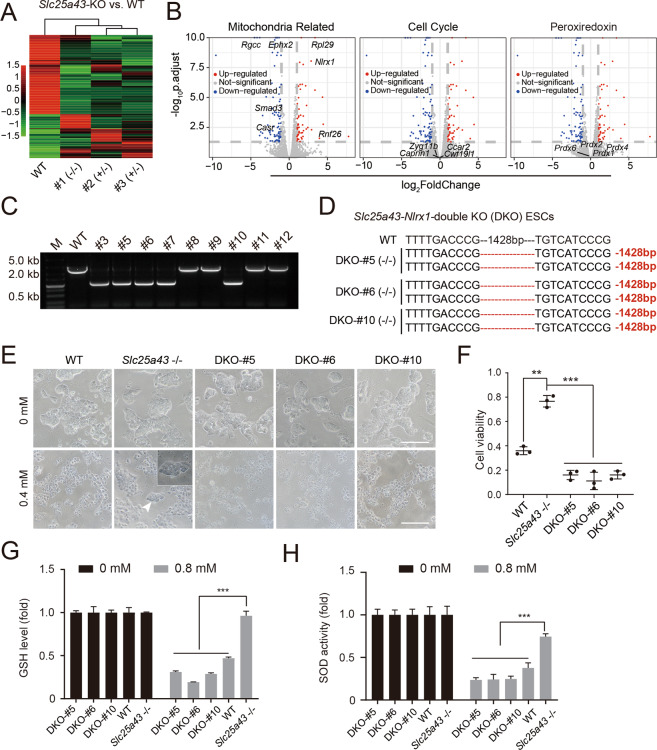
*Nlrx1* is a downstream gene of *Slc25a43* involved in oxidative stress. **A** The heatmap of DEGs among three *Slc25a43*-KO ESCs and WT-diESCs. **B** The volcano plots showed DEGs between *Slc25a43*-KO ESCs and WT-diESCs treated with H_2_O_2_. Highlighted points indicate representative genes related to mitochondrial function, cell cycle and peroxiredoxin. **C** Genotype identification of *Slc25a43*-*Nlrx1*-double KO (DKO) ESCs. **D** Genotyping of DKO ESCs by DNA sequencing. **E** Phase-contrast images of DKO ESCs and WT-diESCs after treatment with 0.4 mM H_2_O_2_ for 4 h. Scale bar, 100 μm. **F** Cell viability of *D*KO ESCs and WT-diESCs treated with 0.4 mM H_2_O_2_ for 4 h. Data represented three independent experiments. *t-*test, **p < 0.01, ***p < 0.001. Data were presented as the mean ± SD. **G** GSH levels in DKO ESCs and WT-diESCs treated with/without 0.8 mM H_2_O_2_ for 4 h. Data represented three independent experiments. *t-*test, ***p < 0.001. Data were presented as the mean ± SD. **H** SOD activities in DKO ESCs and WT-diESCs treated with/without 0.8 mM H_2_O_2_ for 4 h. Data represented three independent experiments. *t-*test, ***p < 0.001. Data were presented as the mean ± SD.

To investigate the mechanism by which *Slc25a43* acted in the context of oxidative stress, we analyzed the global transcriptome levels of *Slc25a43*-KO ESCs and WT-diESCs treated with H_2_O_2_ (0.4 mM, 4 h). There were 895 significant DEGs between *Slc25a4*3-KO ESCs and WT-diESCs (Fig. 5A). According to the GO analysis, the upregulated genes were mainly enriched in defence response, cell proliferation and other functions (Fig. S5A), whereas the downregulated genes were enriched in R-SMAD binding, ROS biosynthetic process and other functions (Fig. S5B). The oxidative stress described previously was correlated with mitochondrial function, [39] the cell cycle [40] and peroxiredoxin [41]. Here, we assessed the relationships of DEGs with these three aspects. There were several oxidative stress DEGs related to mitochondrial function instead of the cell cycle and peroxiredoxin (Fig. 5B). To comprehensively understand the effects of *Slc25a43*-KO, we identified the top 10 upregulated genes (Fig. S5C) and the top 10 downregulated genes (Fig. S5D) among the DEGs. As shown in the list, the significantly upregulated genes (*Rnf26*, *Rpl29* and *Nlrx1*) and the markedly downregulated genes (*Rgcc* and *Casr*) in *Slc25a43*-null ESCs were related to mitochondrial function. To validate whether the downstream genes were involved in oxidative stress, we chose one of the top 10 upregulated genes, *Nlrx1*, to perform validation experiments. NLRX1 is a mitochondrial nucleotide-binding, leucine-rich repeat (NLR)-containing protein that resides in the mitochondria and is encoded by *Nlrx1* [42]. We introduced gene disruptions in *Slc25a43* −/− ESCs by the CRISPR system with specific sgRNAs of *Nlrx1* (Fig. S5E). We identified three *Slc25a43*-*Nlrx1*-double KO ESCs (DKO ESCs) subclones from 12 randomly picked subclones by genotype PCR and sequencing (Fig. 5C, D), which were treated with H_2_O_2_ and analyzed for cell viability. Interestingly, only *Slc25a43* −/− ESCs survived after H_2_O_2_ treatment, whereas all WT-diESCs and DKO ESCs died (Fig. 5E). The CCK-8 analysis further confirmed this observation (Fig. 5F). The major antioxidant assessments showed that the GSH levels and SOD activities of DKO ESCs decreased significantly compared with those of *Slc25a43* −/− ESCs after H_2_O_2_ treatment (Fig. 5G, H). Taken together, these results showed that *Nlrx1* plays a very important role in the antioxidative stress response of *Slc25a43* −/− ESCs.

**Fig. 6:**
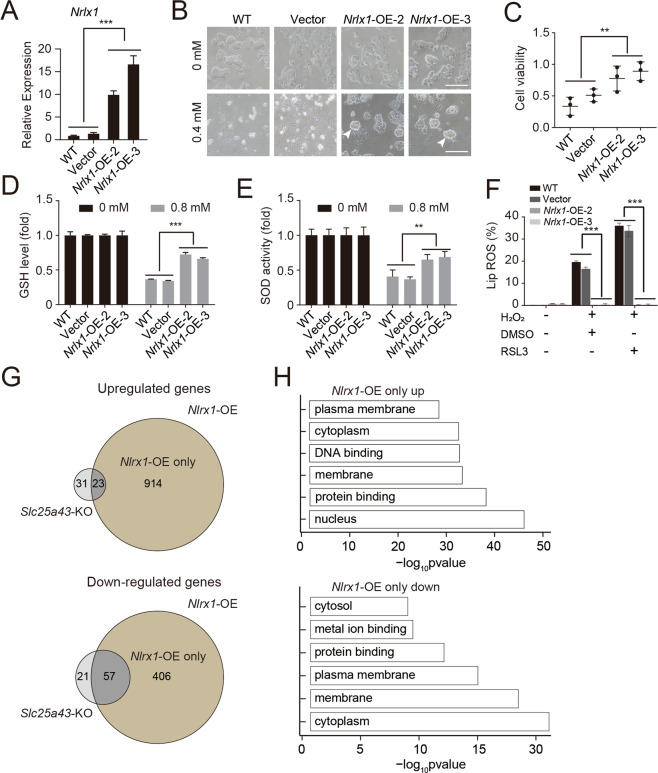
Overexpression of *Nlrx1* suppressed H_2_O_2_-induced oxidative stress. **A** The expression levels of *Nlrx1* in *Nlrx1*-OE-2 ESCs, *Nlrx1*-OE-3 ESCs, empty vector-transfected ESCs and WT-diESCs according to qPCR. The data represented those of three independent experiments (*t-*test; ***p < 0.001). The data were presented as the means ± SDs. **B** Phase-contrast images of *Nlrx1*-OE ESCs, empty vector-transfected ESCs and WT-diESCs after treatment with 0.4 mM H_2_O_2_ for 4 h. Scale bar, 100 μm. **C** Cell viabilities of *Nlrx1*-OE ESCs, empty vector-transfected ESCs and WT-diESCs treated with 0.4 mM H_2_O_2_ for 4 h. The data represented those of three independent experiments (*t-*test; **p < 0.01). The data were presented as the means ± SDs. **D** GSH levels in *Nlrx1*-OE ESCs, empty vector-transfected ESCs and WT-diESCs treated with/without 0.8 mM H_2_O_2_ for 4 h. The data represented those of three independent experiments (*t-*test; ***p < 0.001). The data were presented as the means ± SDs. **E** SOD activities in *Nlrx1*-OE ESCs, empty vector-transfected ESCs and WT-diESCs treated with/without 0.8 mM H_2_O_2_ for 4 h. The data represented those of three independent experiments (*t-*test; **p < 0.01). The data were presented as the means ± SDs. **F** Lipid ROS production in *Nlrx1*-OE ESCs, empty vector-transfected ESCs and WT-diESCs treated with 0.8 mM H_2_O_2_ in the presence or absence of RSL3 (1 μM) for 4 h, as assessed via flow cytometry analysis together with C11-BODIPY staining. The data represented those of three independent experiments (*t-*test; ***p < 0.001). The data were presented as the means ± SDs. **G** Venn diagram of the overlapping genes between *Slc25a43*-KO ESCs and *Nlrx1*-OE ESCs (including upregulated and downregulated genes) upon treatment with H_2_O_2_. The dark gray circle represents the overlapping upregulated DEGs or downregulated DEGs between *Slc25a43*-KO ESCs and *Nlrx1*-OE ESCs. The brown region represented only the upregulated or downregulated DEGs in *Nlrx1*-OE ESCs. **H** GO analysis of only the upregulated and downregulated genes in *Nlrx1*-OE ESCs compared to *Slc25a43*-KO ESCs and WT-diESCs upon treatment with H_2_O_2_.

We were interested in whether *Nlrx1* functions as a modulator of antioxidative stress itself. For this, we overexpressed *Nlrx1* in WT-diESCs and performed subsequent assessments. Exogenous *Nlrx1* was integrated into the genomes of WT-diESCs by a PB system with a GFP indicator (Fig. S6A). The expression levels of *Nlrx1* in *Nlrx1*-overexpression (OE) ESCs were significantly higher than those in WT-diESCs and the empty vector group (Fig. 6A). Compared with the WT-diESCs and the empty vector group members, the *Nlrx1*-OE ESCs survived better after H_2_O_2_ treatment according to observations and CCK-8 analysis (Fig. 6B, C). In addition, the *Nlrx1*-OE ESCs had higher GSH levels and SOD activities than did WT-diESCs and the empty vector group members when treated with H_2_O_2_ (Fig. 6D, E). Overall, *Nlrx1* promoted the resistance of ESCs to oxidative stress. To investigate the mechanism by which *Nlrx1*-OE ESCs suppresses oxidative stress, we treated *Nlrx1*-OE cells with RSL3. However, we did not observe any ROS production increase in *Nlrx1*-OE ESCs when RSL3 was added (Fig. 6F), demonstrating that the antioxidant capacity of the *Nlrx1*-OE cells had no effect on GPX4. To further investigate the potential mechanism underlying *Nlrx1*-OE ESCs, we performed RNA-seq to analyze the global transcriptome levels of *Nlrx1*-OE ESCs, *Slc25a43*-KO ESCs and WT-diESCs when treated with H_2_O_2_ (0.4 mM, 4 h). There were 914 upregulated genes and 406 downregulated genes in the *Nlrx1*-OE ESCs compared to the *Slc25a43*-KO ESCs (Fig. 6G). According to GO analysis, the 914 upregulated genes were mainly enriched in nucleus-related, protein binding and other functions, whereas the 406 downregulated genes were enriched in cytoplasm-related, metal ion binding and other functions (Fig. 6H). These results suggested that a possible reason for the resistance of *Nlrx1*-OE ESCs to oxidative stress involved the regulation of nucleus-related, protein binding, cytoplasm-related and metal ion-binding factors.

## Discussion

In this study, we used haESCs to screen oxidative stress target genes because of their features of unlimited self-renewal ability and single genome phenotype. Abundant homozygous mutations were generated by combining haploid cells with PB transposons (Fig. 2B), which was consistent with previous reports [19]. We identified many genes related to oxidative toxicity from two mutant libraries (ML1 and ML2), which could be used for oxidative stress research. Most of our candidate genes were from the SLC25A family (Table S3). Previous reports showed that SLC25A family proteins were a family of carrier proteins located in the inner membranes of mitochondria that catalyze the translocation of solutes across the membrane [43, 44]. It has been demonstrated that the mitochondrial membrane protein SLC25A43 contributed to cell cycle progression [45, 46], but other functions of *Slc25a43* were unknown. Here, we focused on the effect of *Slc25a43*-null on antioxidative toxicity. Our results showed that KO of *Slc25a43* increased ESC resistance to H_2_O_2_-induced ROS (Fig. 3E–G). The *Slc25a43*-null cells showed higher GSH levels and SOD activities than the control when treated with H_2_O_2_ (Fig. 3H, I), which suggested that KO of *Slc25a43* enhanced the antioxidant capacity. Emerging evidence has shown that various pathological conditions caused by oxidative stress usually caused mitochondrial damage [47]. Our results showed that MMP and ATP production in SLC25A43-null ESCs was higher than that in WT-diESCs treated with oxidants (Fig. 4C, D). Therefore, KO of *Slc25a43* might preserve mitochondrial function. A previous report suggested that *Nlrx1* played a crucial role in ROS-induced oxidative stress [48]. The results of our study demonstrated that *Slc25a43* interacted with *Nlrx1* to exert protective effects on H_2_O_2_-induced cell death. In addition, we found that the expression of *Slc25a43* was decreased in ESCs with increasing passaging, and the cells showed higher antioxidant capacity (data not shown), which was consistent with our results.

In summary, using haESCs, we conducted a forward high-throughput genetic screening of H_2_O_2_ toxicity and identified numerous candidate genes related to the oxidative toxicity response. Furthermore, on the basis of a series of validation experiments, our findings suggested that KO of *Slc25a43* could reduce harm to ESCs caused by oxidants. Moreover, our results indicated that KO of *Slc25a43* protected ESCs from H_2_O_2_-induced cell death through regulation of GPX4 and *Nlrx1*. Furthermore, our data suggested that *Nlrx1* OE protected against oxidative stress. These findings provide new insights into the mechanisms underlying the protection against many pathological processes caused by oxidative stress, including cellular senescence and neurodegenerative diseases [49–51].

## Supplementary information

aj-checklist

SUPPLEMENTAL MATERIAL

Dataset S2

Dataset s3

## Data Availability

The detailed procedures of methods, five figures and the Supplementary Information are attached.
