# Supplementary material for: Genome-wide screening in the haploid system reveals Slc25a43 as a target gene of oxidative toxicity
Source: Cell Death Dis. 2022 Mar 30;13(3):284. doi: 10.1038/s41419-022-04738-4 (PMC8967898; doi:10.1038/s41419-022-04738-4)
Supplement: Supplementary file 2 — SUPPLEMENTAL MATERIAL [file 41419_2022_4738_MOESM2_ESM.docx]

**Title:** Genome-wide Screening in the Haploid System Reveals *Slc25a43* as a Target Gene of Oxidative Toxicity

**Running title:** *Slc25a43*-null Cells Show Oxidative Toxicity Resistance

**Authors and affiliations:**

Jinxin Zhang^1^, Yiding Zhao^1^, Yaru Tian^1^, Mengyang Geng^1^, Yan Liu^2^, Wenhao Zhang^1,3^ and Ling Shuai^1,4^

1 State Key Laboratory of Medicinal Chemical Biology and College of Pharmacy, Nankai University, Tianjin 300350, China.

2 Department of Obstetrics, Tianjin First Central Hospital, Nankai University, Tianjin 300192, China.

3 Chongqing Key Laboratory of Human Embryo Engineering, Chongqing Health Center for Women and Children, Chongqing 400013, China.

4 Tianjin Central Hospital of Gynecology Obstetrics/Tianjin Key Laboratory of Human Development and Reproductive Regulation, Tianjin 300052, China.

**Correspondences:** Yan Liu (30819007@nankai.edu.cn) or Wenhao Zhang (whzhang@nankai.edu.cn) or Ling Shuai (lshuai@nankai.edu.cn)


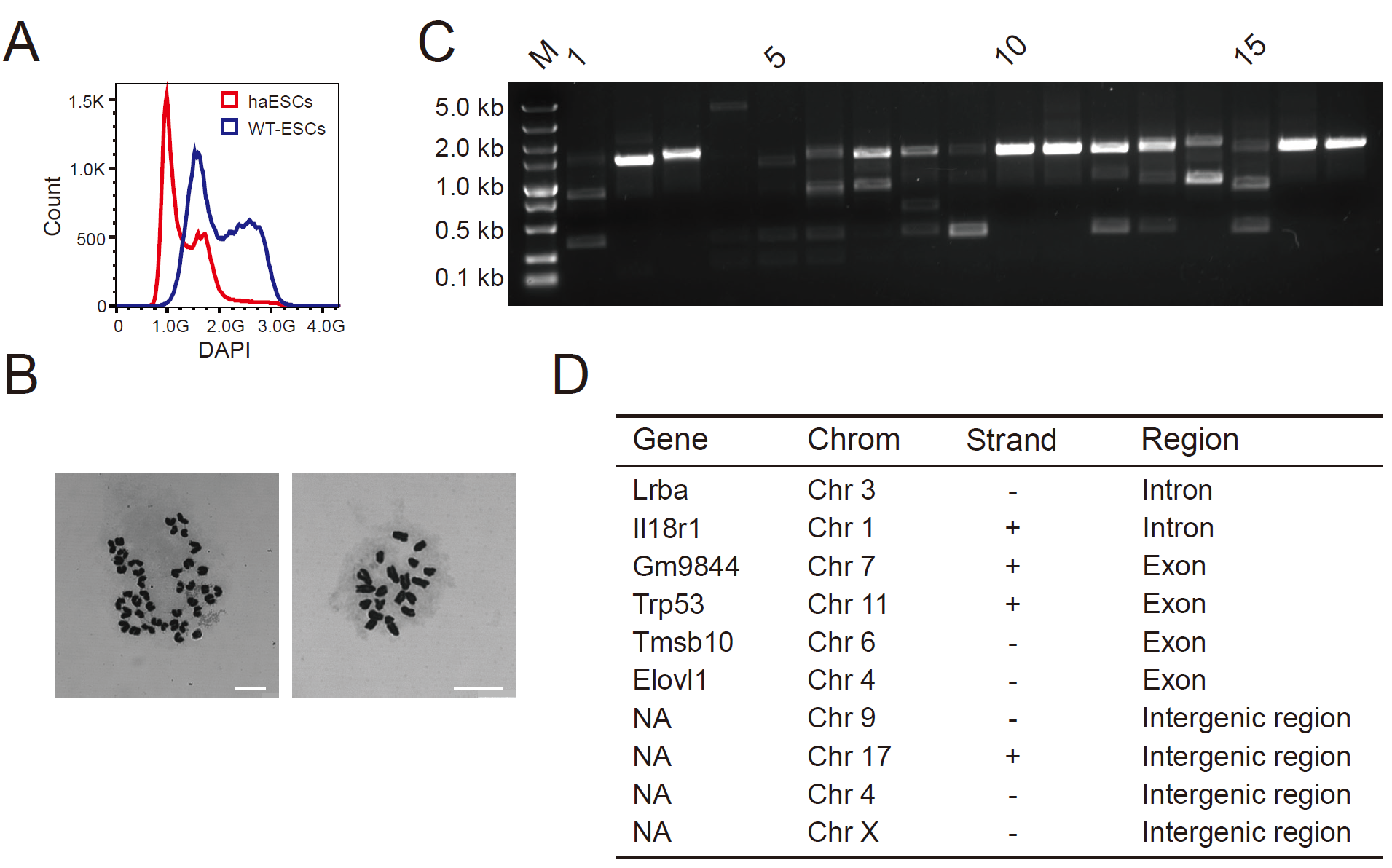


**Fig. S1.** **Analysis of Inserted Genes in Subclones by Inverse PCR**

1. DNA content analysis of mouse haESCs. The percentage of the 1n peak was 20.2%.
2. Chromosome spreads of mouse haESCs and mouse WT-diESCs. Each chromosome spread of haESCs had 20 chromosomes. Scale bar, 10 μm.
3. Inverse PCR analysis of the integration sites in the H_2_O_2_-resistant haESCs. Each lane represented one subclone; each strand indicated a PB integration.
4. Summary of the PB transposon sites in the manually picked subclones identified by inverse PCR.


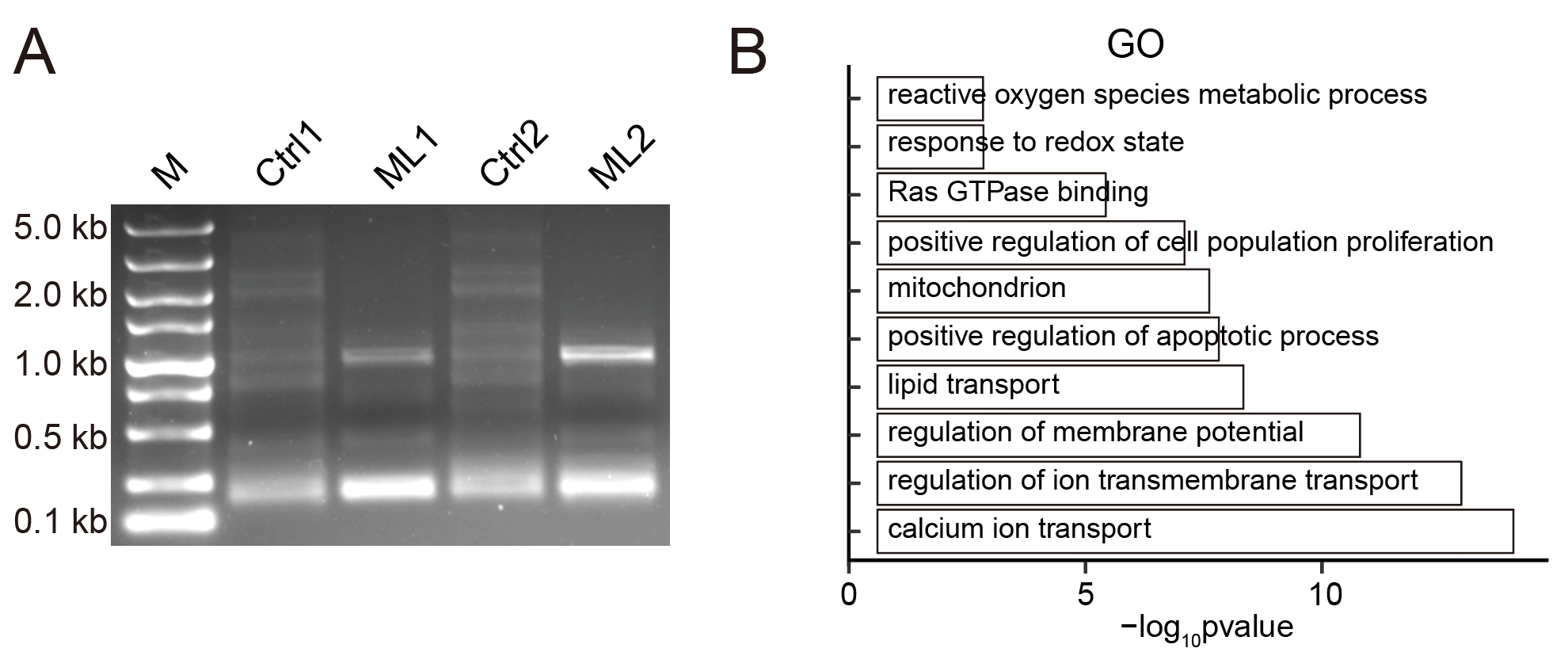


**Fig. S2. Bioinformatics Analysis of Inserted Genes in H_2_O_2_-resistant HaESCs**

1. Splinkerrette PCR analysis of the integration sites in the H_2_O_2_-resistant haESCs. ML1 and ML2 represented two mutant libraries.
2. Gene ontology (GO) analysis of the overlapping top 5000 genes in ML1 and ML2.


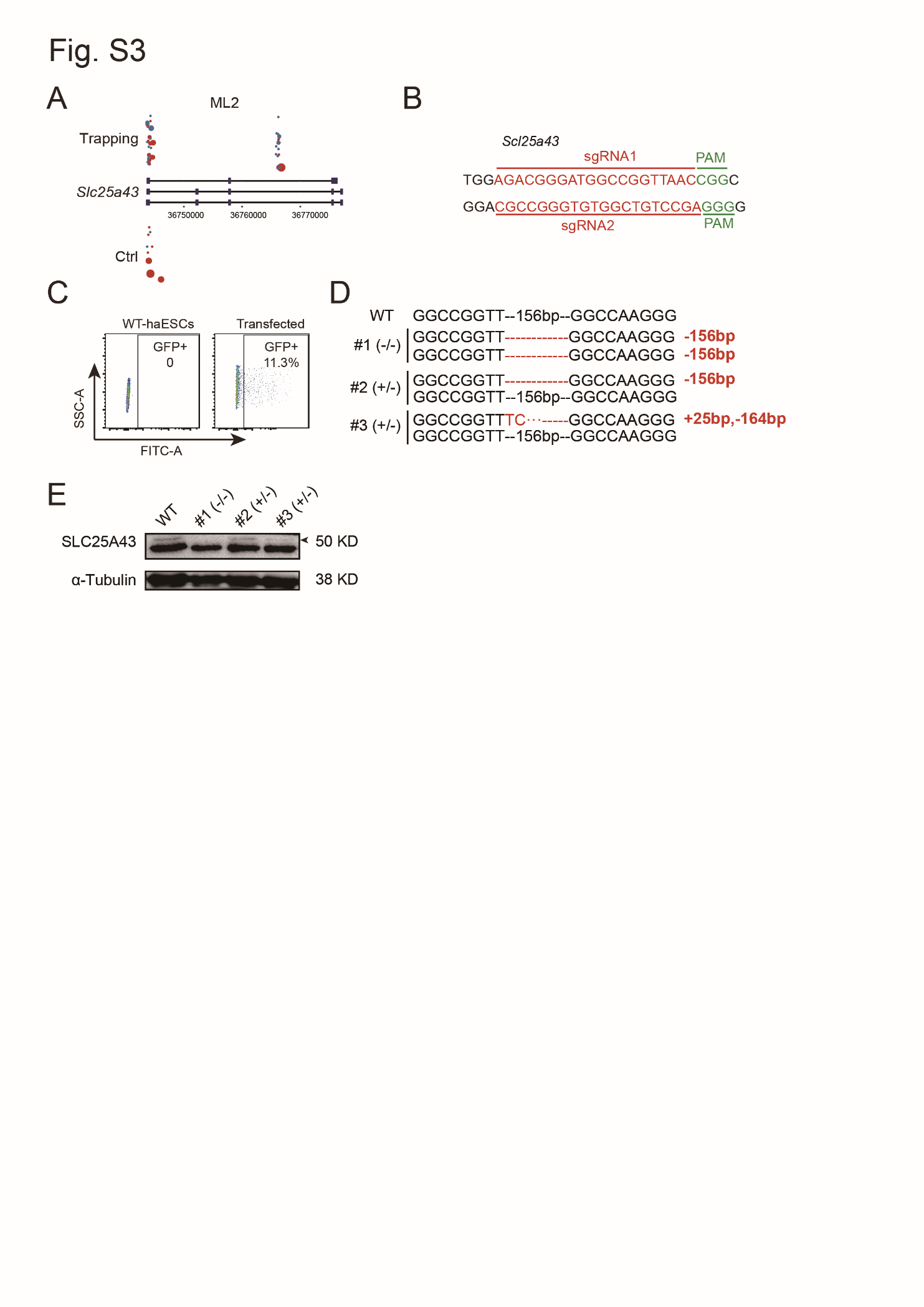


**Fig. S3. Generation of *Slc25a43*-KO ESCs**

(A) Sense (red) and antisense (blue) insertions in *Slc25a43* of ML2. The rectangles indicated the exons, and the size of the circle indicated the insertion numbers.

(B) Specific sgRNAs with CRISPR/Cas9 system to target exon 1 of *Slc25a43*.

(C) Cell sorting of GFP-positive cells 48 h after transfection.

(D) *Slc25a43*-KO genotypes in subclones: #1 (-/-), #2 (+/-) and #3 (+/-).
(E) Western blotting analysis of SLC25A43 (arrowhead indicated) in #1 (-/-), #2 (+/-), #3 (+/-) and WT-diESCs. α-Tublin was used as a loading control.


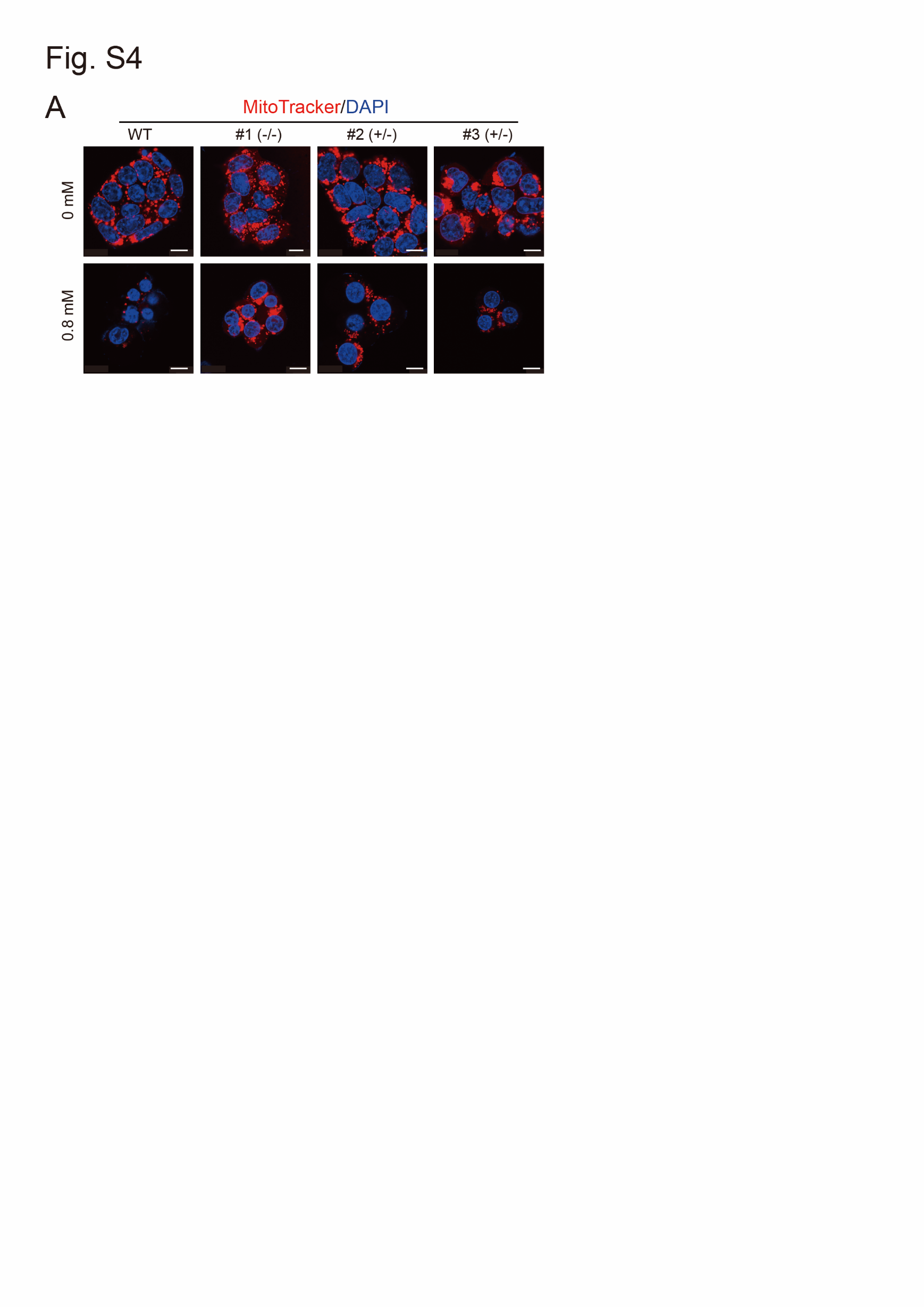


**Fig. S4. Measurement of Mitochondrial ROS in ESCs Treated with H_2_O_2_**

(A) Immunostaining fluorescent images of the mitochondrial ROS by labeling with MitoTracker CMXRos. The *Slc25a43*-KO ESCs and WT-diESCs were treated with/without 0.8 mM H_2_O_2_ for 4 h. Scale bar, 50 μm.


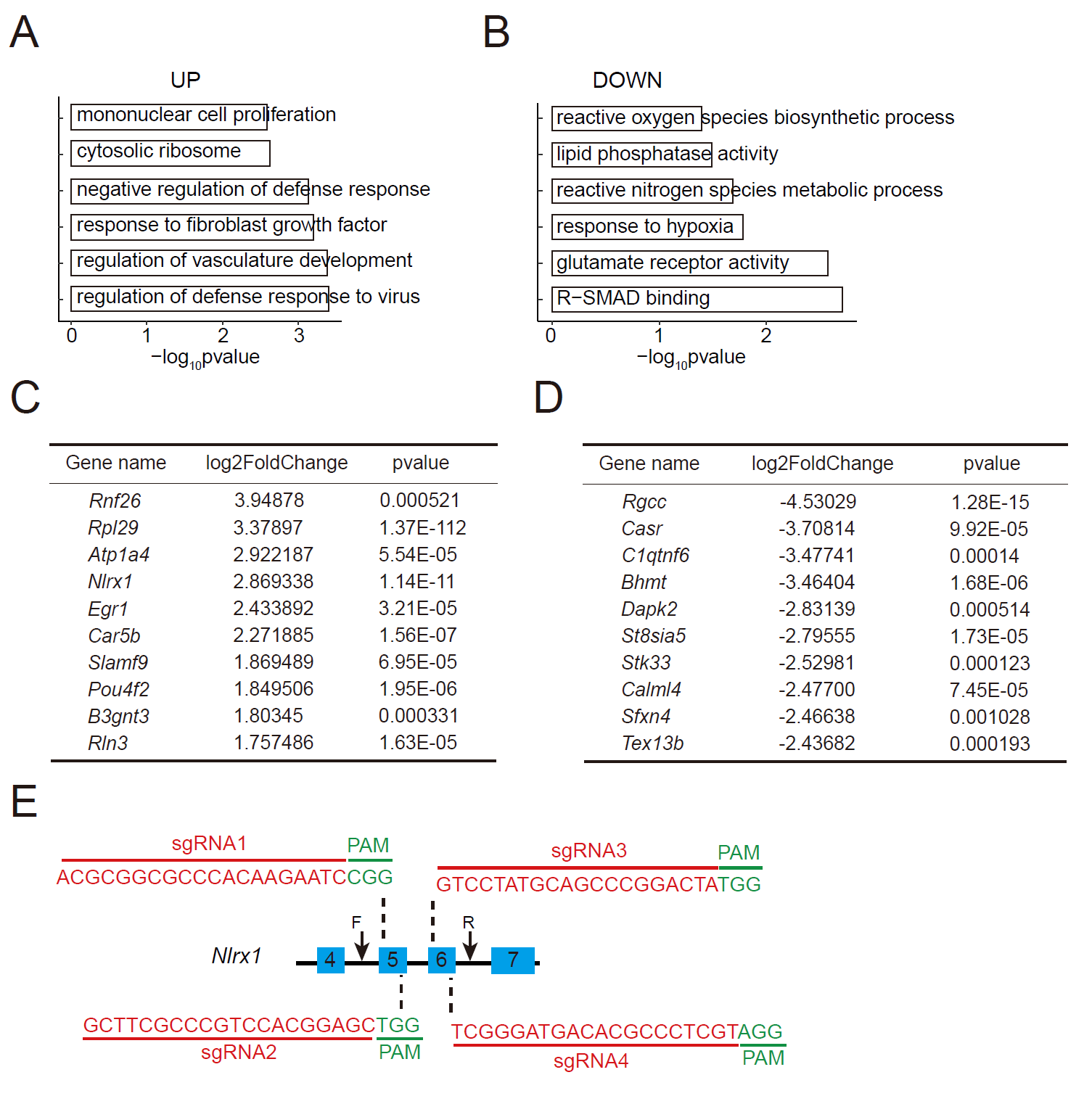


**Fig. S5. Transcriptome of *Slc25a43*-KO ESCs**

(A) GO analysis of the upregulated genes in the *Slc25a43*-KO ESCs with H_2_O_2_ treatment, compared to WT-diESCs.
(B) GO analysis of the downregulated genes in the *Slc25a43*-KO ESCs with H_2_O_2_ treatment, compared to WT-diESCs.
(C) Top ten upregulated genes in *Slc25a43*-KO ESCs, compared to WT-diESCs.

(D) Top ten downregulated genes in *Slc25a43*-KO ESCs, compared to WT-diESCs.

(E) Specific sgRNAs with CRISPR/Cas9 system to target exon 5 and exon 6 of *Nlrx1*.


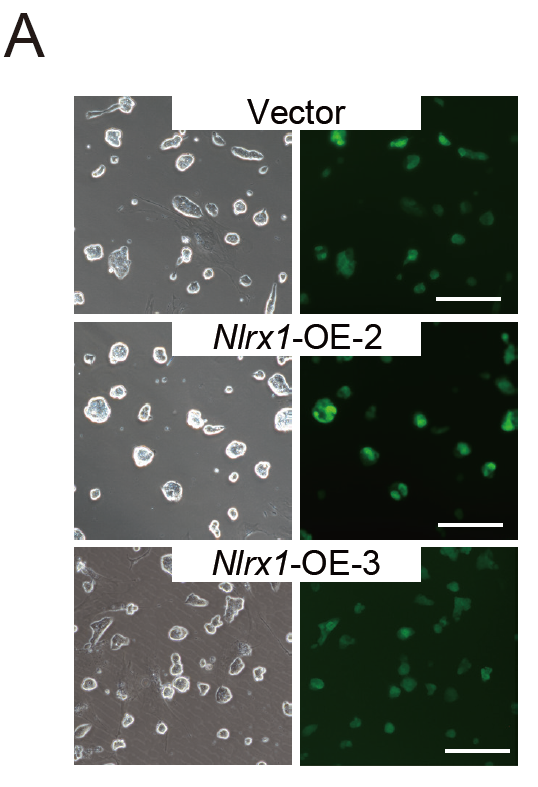


**Fig. S6. Generation of *Nlrx1*-OE ESCs**

(A) Bright-field and FITC images of empty vector transfected ESCs and *Nlrx1*-OE ESCs. Scale bar, 100 μm.

**Table S1. Primer Sequences**

| **Experiment** | **Target** | **Sequence (5’-3’)** |
| --- | --- | --- |
| **Genotype** | *Slc25a43* | F: TGGCCGGACTGCTCTAACT |
|  | *Slc25a43* | R: TTACTTTCGGTAGGCGGCCA |
|  | *Nlrx1* | F: CGTTACCATGGAAACTCGGCA |
|  | *Nlrx1* | R: CCAACACGGCCCACAAATTC |
| **sgRNAs** | *Slc25a43* | 1-1: caccgAGACGGGATGGCCGGTTAAC |
|  | *Slc25a43* | 1-2: aaacGTTAACCGGCCATCCCGTCTc |
|  | *Slc25a43* | 2-1: caccgCGCCGGGTGTGGCTGTCCGA |
|  | *Slc25a43* | 2-2: aaacTCGGACAGCCACACCCGGCGc |
|  | *Nlrx1* | 1-1: caccgACGCGGCGCCCACAAGAATC |
|  | *Nlrx1* | 1-2: aaacGATTCTTGTGGGCGCCGCGTc |
|  | *Nlrx1* | 2-1: caccgGCTTCGCCCGTCCACGGAGC |
|  | *Nlrx1* | 2-2: aaacGCTCCGTGGACGGGCGAAGCc |
|  | *Nlrx1* | 3-1: caccgTCGGGATGACACGCCCTCGT |
|  | *Nlrx1* | 3-2: aaacACGAGGGCGTGTCATCCCGAc |
|  | *Nlrx1* | 4-1: caccgGTCCTATGCAGCCCGGACTA |
|  | *Nlrx1* | 4-2: aaacTAGTCCGGGCTGCATAGGACc |
| **qPCR** | *Gapdh* | F: AGGTCGGTGTGAACGGATTTG |
|  | *Gapdh* | R: TGTAGACCATGTAGTTGAGGTCA |
|  | *Slc25a43* | F: GGCCGCCTACCGAAAGTTC |
|  | *Slc25a43* | R: GCGAGACTTCCAGTCACAATG |
| **Splinkerette PCR** | Adaptor | Top: GTTCCCATGGTACTACTCATATAATACGACTCACT ATAGGTGACAGCGAGCGCT |
|  | Adaptor | Bottom: GCGCTCGCTGTCACCTATAGTGAGTCGTATTA  TAATTTTTTTTTCAAAAAAA |
|  | PB5’ | F1: GATATACAGACCGATAAAACACATGCGTCA |
|  | PB3’ | F1: GACGGATTCGCGCTATTTAGAAAGAGAG |
|  | Adaptor | R2: TAATACGACTCACTATAGG |
|  | PB5’ | F2: ACGCATGATTATCTTTAACGTACGTCAC |
|  | PB3’ | F2: CATGCGTCAATTTTACGCAGACTATC |
| **Inverse PCR** | Left 1 | F: CCTCGATATACAGACCGATAAAACA |
|  |  | R: CAAGGCCTACTAGTATTATGCCCAGT |
|  | Right 1 | F: GGTCATAGGGCCGGGATTC |
|  |  | R: GACTGAGATGTCCTAAATGCACAGC |
|  | Left 2 | F: CATGATTATCTTTAACGTACGTCACAAT |
|  |  | R: GTACATGACCTTATGGGACTTTCCTAC |
|  | Right 2 | F: TCTCCTCCACGTCACCGC |
|  |  | R: GAGCAATATTTCAAGAATGCATGCGTC |
